# Neural Network Model for Detection of Edges Defined by Image Dynamics

**DOI:** 10.3389/fncom.2019.00076

**Published:** 2019-11-07

**Authors:** Patrick A. Shoemaker

**Affiliations:** Computational Science Research Center, San Diego State University, San Diego, CA, United States

**Keywords:** insect vision, visual processing, edge detection, object detection, figure detection, neural networks

## Abstract

Insects can detect the presence of discrete objects in their visual fields based on a range of differences in spatiotemporal characteristics between the images of object and background. This includes but is not limited to relative motion. Evidence suggests that *edge detection* is an integral part of this capability, and this study examines the ability of a bio-inspired processing model to detect the presence of boundaries between two regions of a one-dimensional visual field, based on general differences in image dynamics. The model consists of two parts. The first is an early vision module inspired by insect visual processing, which implements adaptive photoreception, ON and OFF channels with transient and sustained characteristics, and delayed and undelayed signal paths. This is replicated for a number of photoreceptors in a small linear array. It is followed by an artificial neural network trained to discriminate the presence vs. absence of an edge based on the array output signals. Input data are derived from natural imagery and feature both static and moving edges between regions with moving texture, flickering texture, and static patterns in all possible combinations. The model can discriminate the presence of edges, stationary or moving, at rates far higher than chance. The resources required (numbers of neurons and visual signals) are realistic relative to those available in the insect second optic ganglion, where the bulk of such processing would be likely to take place.

## Introduction

It is clear to even a casual observer that many types of insects can detect the presence of discrete objects appearing in their visual fields. While anyone who has tried to swat a fly can attest to its ability to detect and avoid a looming hand, a range of behavioral reactions to visual objects can be found in the insects—which may vary from attractive (as occurs during pursuit of prey or mates, or approach toward objects on which a flying insect intends to land) to avoidant (as with predators or other objects that might collide with the animal). Researchers have studied behavioral reactions to moving visual objects by flies, during both free and constrained flight, in experiments dating back decades. It has often been assumed that the visual motion itself provides the primary cue that is used to discriminate object from background (Götz, 1968; Reichardt and Poggio, 1979; Reichardt et al., 1983, 1989; Egelhaaf, 1985) [While flies can detect moving objects in the presence of simultaneous but differing motion of the visual background, experiments suggest that behavioral strategies, such as head motion, may be used to stabilize the background for periods of time (Fox and Frye, 2014), presumably because this makes object detection easier]. Interestingly, however, it has also long been known that flies can react to stationary, flickering objects (Pick, 1974)—and it is also the case that *motion-blind* flies can still react to moving visual figures (Bahl et al., 2013). While tracking responses to moving objects are generally more robust than to stationary ones (M. Frye, personal communication), I assume for purposes of this study that this represents an attentional effect, and that whatever computational machinery allows insects to detect discrete objects is operative whether those objects are stationary or moving in the animals' field of view.

Recent work with fruit flies has shown that they are in fact capable of performing object detection based on a variety of cues. It has long been established that flying flies exhibit a centering or tracking response to moving, vertically elongate visual features (“bars”) (Reichardt and Wenking, 1969; Poggio and Reichardt, 1973; Reichardt, 1973; Virsik and Reichardt, 1974, 1976);—and they do so whether the bar is analogous to an ordinary moving object (with boundaries and internal visual texture moving with the same direction and speed), or contains only flicker that provides no coherent first-order motion cues—or even when the internal texture moves in the direction *opposite* to motion of the boundaries of the bar itself [a *theta* stimulus (Quenzer and Zanker, 1991; Zanker, 1993)] (Theobald et al., 2008, 2010; Aptekar and Frye, 2013). Fruit flies even display a tracking reaction to stimuli in which a moving bar and the background both flicker, but at different frequencies (M. Frye, personal communication). However, the reactions to these various stimuli are not identical. When the fly is tethered so that it cannot actually center such stimuli, and a bar is moved back and forth periodically in the visual field, then the yaw torque that the fly exerts varies in amplitude and phase depending which class of bar is displayed (Theobald et al., 2010; Aptekar et al., 2012).

Further work has suggested that there are two processing streams in the visual system that contribute to these figure reactions: one that responds to the velocity of the visual texture associated with an object (including coherent motion of the luminance step at its edges), but also a second one that is primarily sensitive to the *position* of the object in the visual field (Aptekar et al., 2012). Such localization could well be implemented by *place-coding* in some portion of the visual pathway that maintains retinotopy—but this presupposes that the object can be *detected* as such by operations prior to or at the level of this place-coding. In order to achieve such detection, it seems clear from the various results just described that flies can rely on a wide range of differences in spatiotemporal statistics to discriminate between “object” and “background.” Furthermore, evidence suggests that object detection is in fact cued by the existence of *edges* or *boundaries* between visual regions with differing spatiotemporal characteristics on the two sides (Aptekar et al., 2017; Keleş and Frye, 2017).

**Figure 1:**
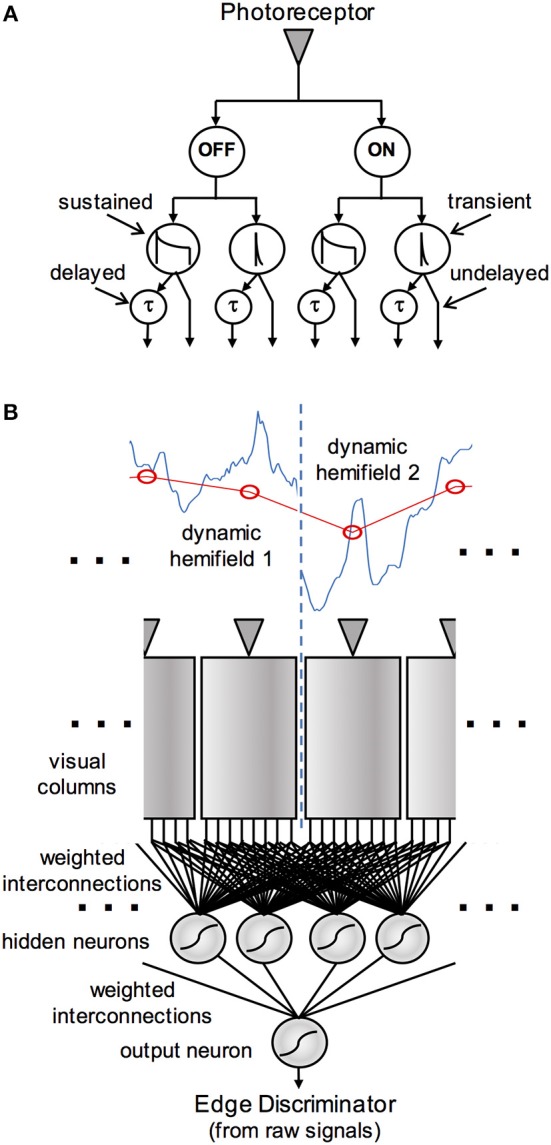
Schematic diagram of the visual processing in an edge detection model. The pathways in **(A)** show the derivation of early vision signals assumed to originate in each visual column. The signal from a photoreceptor (or set of photoreceptors that view the same location in visual space) is split into ON and OFF channels, which are each processed in sustained and transient pathways, and these in turn project via delayed and undelayed pathways to the edge detection network. **(B)** Shows the central part of the visual array, with the photoreceptors stimulated by a low-resolution version (red) of a high-resolution, time-varying image (blue). The eight early vision signals from each column project via weighted interconnections to a “hidden layer” of neural analogs in the edge detection network. The weighted outputs of these model neurons are in turn integrated by a single output neuron.

Detection of *luminance-defined* edges has been an important theme in vertebrate visual neurophysiology for many decades (e.g., Hubel and Wiesel, 1962, 1968) as well as a focus of modeling (e.g., Marr and Hildreth, 1980) – and has also received some attention in the insects (Lehrer et al., 1990). However, to the author's knowledge, the current study represents the first effort to model the detection of *edges defined by differences in spatiotemporal statistics in the imagery on opposite sides*. In it, I consider what sorts of visual signals and processing could support this capability. Inspired by the results of physiological studies (Arnett, 1972; Jansonius and van Hateren, 1991, 1993a,b; Osorio, 1991), I consider a suite of signals in the early visual pathway that encompass ON and OFF responses, both sustained and transient in character. Inspired by models for the detection of visual motion (Hassenstein and Reichardt, 1956; Reichardt, 1961), I model additional processing in the form of delay operators applied to these signals, and “correlations” or products formed pairwise between them. A set of these signals, derived from a small number of neighboring photoreceptors, is used as input to an “artificial neural network” model that is trained to perform edge discrimination based on the values assumed by the signals. A schematic overview of this model is shown in Figure 1. In a set of simulations with dynamic, naturalistic image data, I address the question of whether this model is capable of general edge detection, and if so, how many signals and “neurons” are required for it to be done effectively.

## Materials and Methods

### The Model

The early vision model consists of temporal filters and linear and non-linear mathematical operations, and does not rely on neural modeling *per se*. The artificial neural network that follows is of a conventional “shallow” (two-layer) architecture and performs a stationary input-output mapping (i.e., has no dynamics). All signal representations are continuous-time in nature, although sampled at discrete times for computational purposes.

#### Early Visual Processing

The early visual processing model was implemented to mimic operations believed to reside in the insect retina and the first two optic ganglia, with reference primarily to the dipterans (true flies). These ganglia, the lamina and medulla, are retinotopically organized, each having a distinct column or cartridge of cells for every receptor (or more properly in the flies, each group of receptors that view the same location in visual space), which corresponds to a “visual processing unit” extending through the two ganglia (Laughlin, 1980; Shaw, 1984; Strausfeld, 2008). Although these process visual signals originating in their associated receptors, neurons that extend laterally through multiple columns of cells also provide a mechanism for spatial interactions between visual channels.

Operations akin to spatial high-pass filtering, mediated by lateral inhibitory interactions, are thought to be present in the lamina and the medulla (Zettler and Järvilehto, 1972; DeVoe and Ockleford, 1976; Mimura, 1976). However, preliminary modeling that included such spatial interactions suggested that they had little effect on edge discrimination performance, and so they were omitted in the model used for the study. Following is a description of the elements of this model, which are repeated for each receptor of its simulated vision sensor. Continuous-time representations in the form of differential equations are used to specify temporal filters, but in forms that make clear how they can be discretized in time for numerical integration.

Visual processing can be said to begin with the compound eye optics, which blur the image that is formed on the retina (Zettler and Järvilehto, 1972; Snyder, 1977; van Hateren, 1984) and prevent spatial aliasing. In the model, this is mimicked by spatial convolution of a high-resolution image with a modulation transfer function as the image is downsampled to form the luminance inputs to a simulated photoreceptor array. A Gaussian modulation transfer function is used:

(1) $$\begin{array}{l} MTF=K\cdot \text{exp}(-r^{2}/r_{0}^{2}), \end{array}$$

where *r* represents distance (i.e., viewing angle) from the central location being sampled, and the space constant *r*_0_ is set so that the width of the Gaussian function at half-maximum is equal to 1.4 times the inter-receptor viewing angle. In practice, this function defined with finite support over a region of extent ±2*r*_0_. *K* is a normalizing constant (set such that the sum of *MTF* over this region is unity). This spatial convolution and downsampling occur at every time step of a dynamic simulation.

Much as in vertebrates, insect photoreceptors are non-linear and adaptive in response to impinging light. Although in the flies, multiple receptor cells view the same location in visual space, I model these with a single receptor unit whose characteristics can be described with an adaptive Lipetz (Lipetz, 1971) or Naka-Rushton (Naka and Rushton, 1966) function:

(2) $$\begin{array}{l} U=I^{\text{λ}}/(I^{\text{λ}}+I_{0}^{\text{λ}}), \end{array}$$

where *U* is output, representing modulation of post-synaptic elements (ganglion cells) by the receptor unit; *I* is input (luminance), the parameter λ = 0.7 is called the Lipetz exponent, and *I*_0_ is an adaptive state corresponding to a temporally filtered version of *I* (with a linear first-order low-pass filter):

(3) $$\begin{array}{l} dI_{o}=(dt/{\text{τ}}_{U})\left(I\;-\;I_{0}\right), \end{array}$$

where τ_*U*_ = 750 ms is the time constant for photo-adaptation. The receptors thought to be involved in motion vision have peak sensitivity in the green (Srinivasan and Guy, 1990), and green sensitivity was implemented in this model as well, by using only the green color channel in the input imagery.

Light-sensitive neurons in the lamina, which follows the retina in the visual pathway, show evidence of high-pass temporal in addition to spatial filtering (Laughlin, 1976, 1984). The Lamina Monopolar Cells (LMCs) respond transiently to changes in luminance, and in addition they may play a role in subsequent segregation of visual signals into ON and OFF channels: blocking of one class of LMC (L1) results in a loss of responsiveness of downstream motion-sensitive neurons to moving ON edges, whereas blocking of a second class (L2) causes a loss of responsiveness to OFF edges (Joesch et al., 2010). In the medulla, cells are found with physiologically-identifiable responses to one or the other polarity of luminance change (Strother et al., 2014). Interestingly, extracellular recordings of fibers connecting the lamina and medulla show the presence of highly transient, *full-wave*-rectified responses to luminance changes (Arnett, 1972; Osorio, 1991; Jansonius and van Hateren, 1993a; Wiederman et al., 2008). Other fibers in the same tract show transient initial phases that also have a sustained component in response to luminance increases (Arnett, 1972; Jansonius and van Hateren, 1993b). In light of this evidence, I postulate that ON and OFF channels with both transient and sustained response elements are present in the medulla, and model them as follows.

Photoreceptor output signals are initially passed through a linear, first-order temporal high-pass filter and this signal is half-wave rectified for both positive and negative phases, with the negative phase inverted, in order to form positive-going ON and OFF channel signals *V*_*ON*_ and *V*_*OFF*_:

(4) $$\begin{array}{l} dV_{f}=dU-(dt/{\text{τ}}_{R})V_{f} \end{array}$$

(5) $$\begin{array}{l} V_{ON}=0.5(|V_{f}|+V_{f})\\ V_{OFF}=0.5(|V_{f}|-V_{f}) \end{array}$$

where *U* is the photoreceptor output signal from Equation (2), *V*_*f*_ is the filtered signal, *V*_*ON*_ and *V*_*OFF*_ are respectively the on and off channel signals, and τ_*R*_ = 200 ms is the high-pass time constant.

Subsequent operations are repeated for each of the ON and OFF signal channels, and the ON and OFF subscripts will hereafter be omitted from the variable names (e.g., *V* will be used to represent either *V*_*ON*_ or *V*_*OFF*_).

To form sustained outputs, the initial rectified signals are each processed with a “relaxed” temporal high-pass filter that passes 40% dc, and then half-wave rectified for the positive phase:

(6) $$\begin{array}{l} dW_{f}=dV+(dt/\tau_{S})(0.4V-W_{f}) \end{array}$$

(7) $$\begin{array}{l} W=0.5\left(|W_{f}|+W_{f}\right), \end{array}$$

where *W* is the sustained signal for the ON or OFF channel, and τ_*R*_ = 50 ms is the time constant for the relaxed high-pass filter.

Transient outputs are formed in parallel with sustained outputs using the “Rectifying Transient Cell” model of Wiederman et al. (2008). The ON and OFF signals from Equation (5) each are passed through a first-order high-pass filter and half-wave rectified:

(8) $$\begin{array}{l} dX_{f}=dV-(dt/{\text{τ}}_{A})X_{f} \end{array}$$

(9) $$\begin{array}{l} X_{r}=0.5\left(|X_{f}|+X_{f}\right), \end{array}$$

where τ_*A*_ = 40 ms is the time constant for the high-pass filter. “Adaptive states” are then computed from these signals using a non-linear “fast depolarization, slow repolarization” filter (details in Wiederman et al., 2008):

(10) $$\begin{array}{l} dX_{a}=(dt/{\text{τ}}_{Ad})\left(X_{r}-\;X_{a}\right)\;if\;X_{r}-\;X_{a}\geq 0\\ dX_{a}=(dt/{\text{τ}}_{Ar})\left(X_{r}-\;X_{a}\right)\;if\;X_{r}-X_{a}<0, \end{array}$$

where *X*_*a*_ is the adaptive state, the “depolarization” time constant τ_*Ad*_ = 2 ms, and the “repolarization” time constant τ_*Ar*_ = 100 ms. The adaptive state is then subtracted from each signal, and the results are half-wave rectified for the positive phases:

(11) $$\begin{array}{l} X=0.5\left(|X_{r}-X_{a}|+X_{r}-X_{a}\right), \end{array}$$

where *X* represents the transient signal for either the ON or OFF channel.

The output amplitudes of the sustained and transient signals are then normalized so that their standard deviations during positive excursions are each unity over the visual input dataset described below.

Finally, a delay operator modeled as the phase delay of a first-order temporal low-pass filter is also applied to the ON and OFF, transient and sustained signals, to generate four additional output signals:

(12) $$\begin{array}{l} dX_{d}=\left(dt/\tau_{D}\right)\left(X-X_{d}\right) \end{array}$$

(13) $$\begin{array}{l} dW_{d}=\left(dt/\tau_{D}\right)\left({W-W}_{d}\right), \end{array}$$

where *X*_*d*_ represents the delayed sustained signal and *W*_*d*_ the delayed transient signal for either the ON or OFF channel, and τ_*D*_ = 50 ms is the delay time constant.

There are thus eight primitive early vision signals per visual processing unit, which are used for purposes of edge detection. These are replicated for multiple adjacent processing units, which view adjacent bits of an input image.

For generality, I allow for the formation of products or “correlations” between all pairwise combinations of these eight signals for each processing unit, excluding simple square terms. This results in 28 products for each receptor. In addition, I allow spatial interactions in the form of products between all possible combinations of signals between adjacent processing units, for a total of 64 more products per pair. Naturally, any information that is present in these correlations is also present in the primitive signals from which they are derived, but the motivation for including them is that certain of the products may represent that information in a form that is better suited for edge discrimination—for example, since such products have been used to model visual motion detection.

#### Edge Detection Mechanism

Edge detection is performed by an “artificial neural network” (ANN)-style model, in which selected outputs from early vision project to a “hidden layer” of neural analogs, as depicted in Figure 1. These outputs may include the eight early vision signals, or the correlator signals, as described in section Early Visual Processing above. The hidden layer nominally contains 32 units, and is fully connected, meaning that each neuron receives weighted inputs from the entire set of vision signals. The hidden layer neurons project their outputs via a second set of weights to a single output neuron that codes for either the presence or the absence of an edge in a scene by its activation or lack thereof, respectively. Interconnection weights are allowed to be either positive (to model excitatory synaptic input) or negative (for inhibitory synaptic input) in sign, with magnitude representative of synaptic efficacy. A constant offset term or “bias,” corresponding to a soft activation threshold, is added to the weighted sum that forms the input to each neuron, and this quantity is passed through a logistic activation function to form the neural output; i.e.,

(14) $$\begin{array}{l} O_{i}=1/\left[1+\exp\left(-\left(\Sigma_{j}W_{ij}I_{j}+b_{i}\right)\right)\right] \end{array}$$

where *i* and *j* are used to index neurons and their inputs, respectively, *O*_*i*_ is the output of the *i* ^*th*^ neuron, *I*_*j*_ is its *j* ^*th*^ input, *W*_*ij*_ are the synaptic weights, and *b*_*i*_ the bias.

The weights and biases in this network are the parameters that determine how it classifies visual scenes. These are acquired by a stochastic approximation learning procedure, implemented by error back-propagation using a sum-squared-error loss function (Rumelhart et al., 1986). If analogous interconnections are in fact hard-wired in an insect brain, this training can be regarded as mimicking the effect of millions of years of evolutionary pressure on the acquisition of function in the relevant part of the nervous system.

#### Visual Array and Input Scenes

Experiments were performed using a one-dimensional model array of either six or eight co-linear photoreceptors, with an inter-receptor viewing angle of ~1°, and associated visual processing units, followed by the edge detection network. An *edge* in this context is defined as a boundary between two segments of the one-dimensional visual field that have different image dynamics. This study thus models the detection of edges of the minimum possible lateral extent. Outputs from either the two, four, or six most central of the visual units are passed as inputs to a conformal ANN for detection of edges that are present between the two center receptors (The corresponding total number of primitive early visual signals for these three cases is 16, 24, or 32, respectively, and of correlation signals is 120, 304, or 488, respectively). The edge discrimination ANN is trained with imagery that either has an edge between the two center receptors, or no edge within the visual field.

### Simulation Methods

#### Simulations of Visual Edge Detection

Time-domain simulations were performed to compute the response of the early vision model to visual stimuli. Stimuli comprised either six or eight time-varying luminance signals, which were passed through the optics model, and which comprise the inputs to the photoreceptor array. These were generated according to various scenarios described in section Generation of Visual Data, and stored for processing with the vision model.

Because this study focuses on edge detection based on differences in spatiotemporal statistics, it was conducted primarily with scenarios where the edges themselves are *stationary* and placed at the center of the photoreceptor array (This also facilitates computationally-tractable generation of large volumes of data). However, a smaller volume of input data with *moving* edges was generated as well. Input data for scenarios without edges, corresponding to retinal imagery with consistent dynamics across the entire array, were also generated. Eight inputs to eight receptors were generated for the fixed-edge scenarios (to allow for evaluation of lateral spatial inhibition in the early phase of the effort), and six for the moving-edge scenarios.

The response of the visual processing model to the generated stimuli was computed by numerical integration (simple quadrature) with a time step of 200 μs (i.e., one-tenth the smallest time constant in the model). Output signals were averaged over 10 ms periods, and the resultant series of these averages stored as outputs for each scenario simulated. For the fixed-edge and no-edge scenes, the 10 ms periods were successive and outputs stored over the entire duration of a simulation. In the moving-edge scenes, edges cross the entire input array but output data were only stored while an edge was passing between the two central detectors in the array. The 10 ms averaging periods in these cases were overlapped by 50% to increase the number of available data.

The averaged vision system outputs were subsequently recalled and passed one at a time through the edge detection network, which produces a single output in response to each.

#### Generation of Visual Data

The classes of image dynamics that were used include internal motion to the left or to the right at one of three different speeds (corresponding to 25°/s, 50°/s, or 100°/s in the input images); flicker at one of three mean temporal frequencies; and static luminance patterns (a static input to a photoreceptor results in outputs of zero from its modeled visual processing unit, so that discrimination of static subimages from time-varying ones should be trivial—except for the fact that there is some “spillover” of time-varying luminance from a dynamic region to receptors viewing a static region, due to optical blurring).

**Figure 2:**
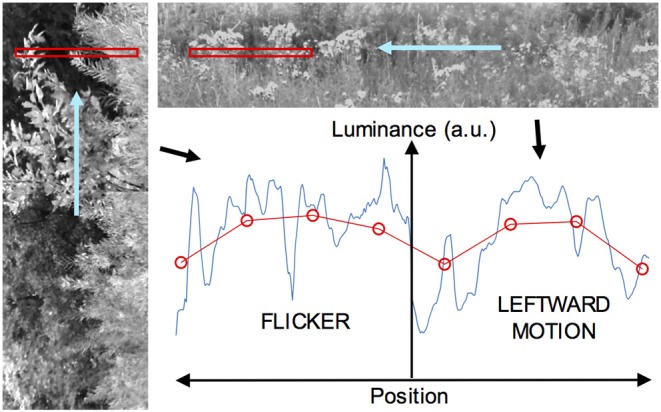
Derivation of dynamic image data, for the case of a visual field with a flickering image on one side and a moving image on the other. On the left, a vertically-oriented image (the green channel in a color image, here rendered in grayscale) is animated in the upward direction so it moves across a horizontal window, shown in red, from which luminance data are sampled at each time step. This yields a one-dimensional flickering subimage. At upper right, a horizontally-oriented image is moved right to left across a horizontal window to give a leftward-moving subimage (More than a single row of pixels are shown for clarity). The two subimages are butted together to form a complete image with a central edge. The graph at lower right depicts such an image sampled at a particular time, with the original high-resolution data shown in blue, and the blurred and downsampled data for the visual array in red. The ordinate represents light intensity (in arbitrary units) and the abscissa space in this plot.

The data used to form these inputs were derived from photographic imagery, obtained from six digital photographs of predominantly natural scenes. As noted, only the green channel (with 8 bits resolution per pixel) was used. During this process, contiguous blocks of pixel data were extracted from images selected at random, and from locations within an image also selected at random. Blocks that had more than 15% of pixel values at saturation (values of 0 or 255) were rejected. For static stimuli, blocks consisted of a portion of a single row of pixels in a photo. Moving and flickering imagery were generated by animation, as illustrated in Figure 2. For moving stimuli, a long one-dimensional image was formed by concatenation of many blocks from single rows (although this introduces luminance discontinuities, these are common in natural imagery), and then animated horizontally so that it moved across a window corresponding to the visual field or part thereof viewing the stimulus. Flickering stimuli were implemented with motion *perpendicular* to the axis of the receptor array, as follows. Two-dimensional blocks of pixels were extracted from the photographs and the visual receptive field or part thereof was filled using *columns* of this data—i.e., the horizontal axis of the image was oriented vertically with respect to the receptor array, as depicted in Figure 2. Many such blocks were concatenated and then animated in the vertical direction, at one of the three speeds used for the moving image data. The corresponding receptor signals do not have a cross-correlation peak at the receptor transit time as is present when the motion is axial.

For stimuli with static edges, a window containing imagery of a particular dynamic class corresponded to a fixed half of the visual field. For stimuli with moving edges, the windows themselves were animated. For a “Fourier” stimulus, corresponding to an ordinary moving object, the window moved in the same direction and at the same speed as the internal visual texture. For a theta object, the texture moved in the opposite direction but same speed as the window.

For all stimuli that contained dynamic images, the raw imagery was upsampled with linear interpolation such that the spacing between the higher-resolution pixels corresponded to the distance traveled by the moving imagery in one integration time step. All retinal imagery, including images with edges, was assembled with this high-resolution data at each time step, and then downsampled with blurring by convolution with the (one-dimensional) MTF, in order to generate the receptor input data. In this way, any edge present in an image was smoothed as it would be by compound eye optics. The selection of this downsampling ratio with respect to the original photographs yielded the inter-receptor viewing angle of around 1°.

With this methodology, sequences of input data for scenarios with fixed edges were created for every possible pairwise combination of leftward motion, rightward motion, and flicker, in each possible position (left or right half of the visual field), for each of the three animation speeds. This yields 18 different classes of edge scenarios. The medium animation speed (50°/s) was represented twice as often as the slow or fast speeds in these data. In addition, inputs were created for all combinations of static imagery with the three dynamic image classes, in each possible position, and with animation of the dynamic hemi-field at the medium speed, for six additional edge scenarios. These were represented with frequency equal that for the medium-speed scenarios with the dynamic image pairs. In addition, full-field (no-edge) input data were also generated for each of the three dynamic image classes, such that each class was represented as frequently in these data as in the edge scenarios. Inputs for the full-field static case were not generated from images since the visual outputs in response to such scenarios are zero. Sufficient input data were generated for the static-edge stimuli to produce a total of 72,000 output data (for each of the raw and correlator signal sets) from the visual processing model. These were combined with an equal number of data from full-field stimuli, to comprise a sample from a visual universe in which the prior probability of viewing an edge is 50%. This corpus of data corresponds to a total of 1440s of responses to the various stimulus classes.

In the scenarios with moving edges, all edges moved left to right at 50°/s and all imagery was animated at the same speed. Flickering and static imagery were represented along with theta and Fourier objects, in every combination, and with imagery of each type appearing with equal frequency on each side of the moving edge. In a particular animated sequence, a succession of edges between two different image classes moved continuously across the receptive field, such that each class appeared alternately to the left and to the right of the edge. A total of 20,000 output data were produced for the moving-edge stimuli (all recorded while edges were passing between the two central detectors in the array), and paired with 20,000 outputs from the full complement of full-field stimuli to yield a corpus of 40,000 data, in this case for the raw vision signals only. Examples of two classes of edged stimuli, one with a stationary edge and the second with a moving edge, are shown in Supplementary Movies 1, 2.

#### Neural Network Implementation and Training

The ANNs were implemented each neuron according to Equation (14), using table lookup for the activation function and its derivative. Initial weight and bias values were chosen at random according to a Gaussian distribution with standard deviation $\sigma =I_{s}/\left(1.33\cdot \sqrt{N}\right)$, where *I*_*s*_ is the index into the lookup tables corresponding to an argument of unity, in this case assigned a value of 200, and *N* is the number of hidden neurons. The weighted sums in Equation 1 were consequently rounded to integer values when “neural” responses were computed, in order to give indices into those tables.

Networks were trained with a target output of 1 if an edge were present in an input datum, and 0 if not. Minimization of the sum-square-error loss function with these target outputs trains a network to estimate the *posterior probability* that an input pattern belongs to the “edge” class (Shoemaker, 1991). Therefore, in order to score its detection capability, the network was treated as a Bayes classifier: an input pattern was assigned to the “edge” class if the response of the output neuron was >1/2, and to the “no-edge” class if not, and this assignment compared to the true class membership. Scores were compiled over entire training or test datasets to monitor overall performance. The expected score for random class assignment is 50% correct, since the decision is binary.

**Figure 3:**
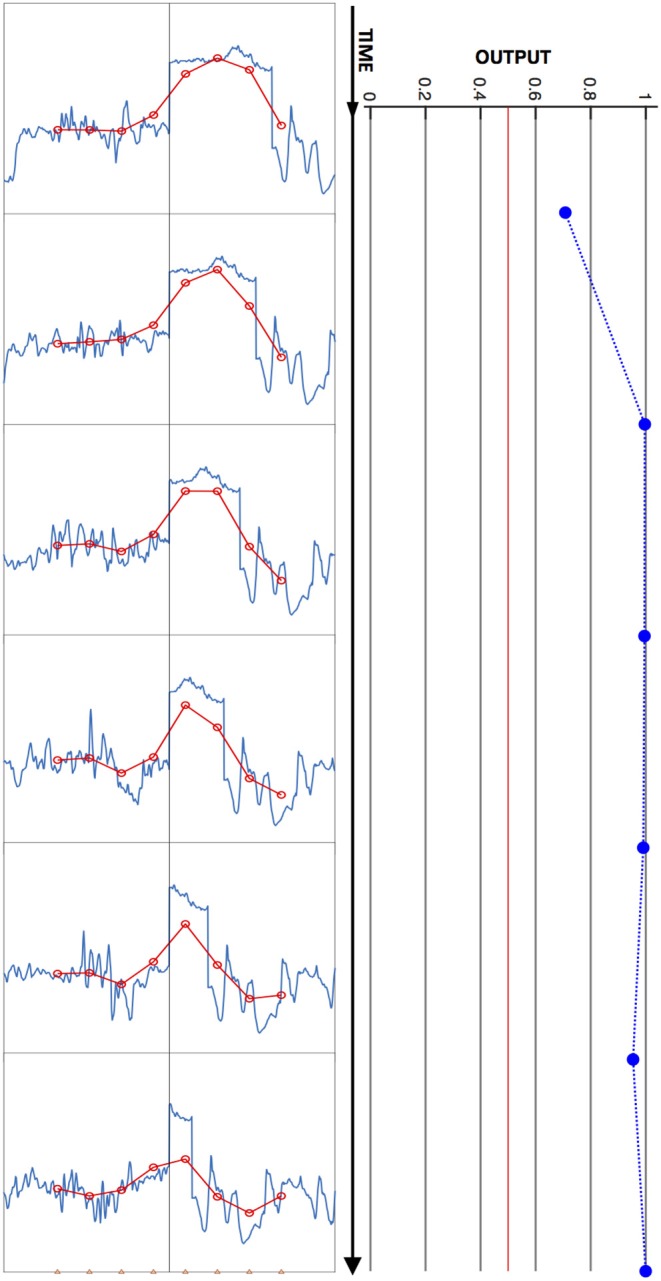
Model input/output relation illustrated. A time series of input images is shown at left, and time-aligned network output values at right, with time running from top to bottom. Each plot depicts a one-dimensional high-resolution image (blue) and corresponding blurred, downsampled (red) image presented to the network, as in Figure 2. In this stimulus sequence, the left hemifield contains a flickering sub-image, and the right, a leftward-moving subimage. The time interval between plotted output data is 10 ms. The network output remains above the Bayes decision threshold of ½ (vertical red line) for this snippet of stimulus, and is therefore scored as correctly indicating the presence of an edge throughout.

An example of an input-output mapping performed by this model is illustrated in Figure 3. This figure shows a short series of visual input patterns, with two dynamic hemifields and a fixed edge between them, as well as the outputs they elicit from a trained network at 10 ms intervals.

The input data for any regimen were divided with ¾ used for training and ¼ held out to test performance. Learning consisted of a conventional iterative process, conducted in *epochs* during which the network parameters were adjusted according to gradient descent for every input datum in a training set. These data were chosen in random order by random permutation of their indices before each epoch. The learning rate η (i.e., the constant that multiplies the gradient of the objective function) was reduced after each epoch according to a hyperbolic function of the epoch number: η = η_0_/(1 + η_*e*_*k*_*e*_), where *k*_*e*_ is the epoch number and the parameters η_0_ and η_*e*_ assumed values in the ranges of 0.75–2.0 and 0.005–0.01, respectively. When a network was trained *de novo*, the process was typically initiated with training from five different random parameter choices for 50 epochs each, and then continued for an additional 750–950 epochs with the best-performing of the five weight sets, for a total of 800–1,000 epochs. During the development of this procedure, multi-start optimization (training runs initiated from different random initial conditions) and simulated annealing (by periodic increases in the otherwise-decaying learning rate η) were used to test for local minima in the loss function. In general, a sample of five different networks was generated by training from five different parameter initializations for each of the input datasets and architectures described below.

Networks were trained for the fixed and the moving-edge cases with the entire training data corpus for each described in the prior section. Training was repeated for three different receptive field sizes, with inputs drawn from the central two, four, or six visual units. This was carried out for both raw vision signals and product terms for the fixed-edge data, but only the raw signals in the moving-edge case.

In some experiments, learning was also carried out with subsets of the fixed-edge data encompassing pairwise combinations of just two image classes. These used raw vision outputs from the central four visual units only. The two image classes were equally represented on either side of the edge, and the three animation speeds present in the dynamic data in the same proportion described above. The full-field data in these experiments included all image classes, but in the same total number as the edge data in order to maintain equal prior probabilities. These networks were thus trained to distinguish a single type of edge from any class of full-field stimulus. Their training proceeded from an initial weight set determined by learning with the full training set.

Finally, some networks were trained with *both* fixed-edge and moving-edge data. In these experiments, the complete corpus of moving-edge data was augmented with an equal-sized subset of the fixed-edge data, one in which all possible edge scenarios were represented. The full-field data were similarly doubled in number in this training set. These experiments were repeated for all three receptive field sizes, but used raw vision signals only.

In further experiments, fixed-edge networks were ‘pruned', i.e., the number of inputs or hidden-layer neurons systematically reduced from their nominal values. During this process, individual input components or hidden neurons in a trained network are tested for significance by zeroing their signals out one by one and evaluating the sum-square error loss function over the entire training set. The input or neuron whose omission results in the smallest increase in the loss function is removed permanently, including any weight or bias parameters associated with it. The network is then re-trained for 100–200 learning epochs, and the process repeated until a single input or hidden neuron remains.

#### Tools

The study was conducted using software written in MATLAB (MathWorks, Natick, MA, USA).

## Results

The results of the study demonstrate the capabilities of the model for edge discrimination, and the relative consistency with which function can be acquired by the stochastic gradient descent learning method. Robust results are obtained with the training procedures described in the previous section: classification score histories appear to approach asymptotic values by the end of training, and multi-start runs and simulated annealing did not reveal the existence of local loss function minima with significant differences in classification performance.

### How Well Can the Model Detect Edges?

**Figure 4:**
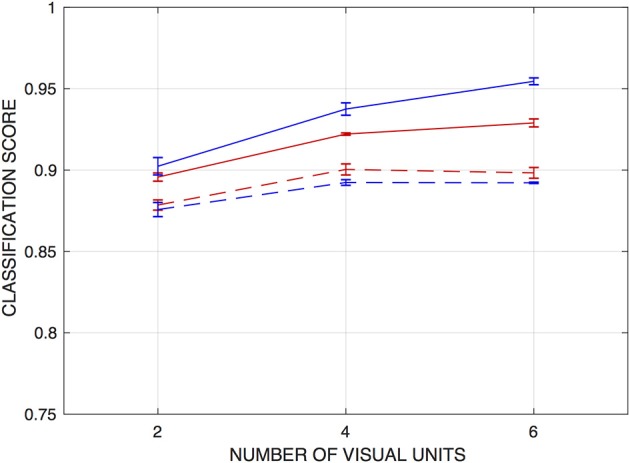
Classification scores for networks trained to detect fixed edges, as functions of the number of visual units from which inputs are drawn. Scores are the fraction of input patterns correctly assigned. Red curves are for networks operating on raw visual signals, and blue curves for networks operating on “correlations” or product signals. Solid lines indicate performance on the training set, and dashed lines on a holdout set. Error bars indicate ± one standard deviation (over *n* = 5 independently-trained detection networks).

For the ANNs trained on the fixed-edge training data, networks that operate on the raw vision signals *or* on the correlator outputs from just the two central visual processing units can achieve scores of around 90% correct. Accuracy improves modestly when signals from nearest and next-nearest neighboring visual units are included in the training inputs. Figure 4 depicts this performance as a function of the number of visual units from which inputs were drawn.

Classification performance on the sets of test inputs is also shown in Figure 4, and these scores suggest that some degree of overtraining occurs with the network architectures used, in spite of the size of the training set. In particular, test scores for the networks that operate on the correlator outputs, which have an order of magnitude more parameters than those operating on the raw vision signals, were some 3.5–6% lower than for the training set (and marginally lower than networks operating on the raw vision signals). The results also suggest that the products do not present essential information in a way that facilitates edge detection by this approach. As with the training set, there is a modest increase in classification accuracy when inputs are drawn from four visual units rather than two—but there is no advantage to expanding this range to six units.

As might be expected, when networks were trained with single fixed-edge types, classification scores on those edge types exceeded scores for the networks trained on the entire training set. For edges between dynamic hemi-fields, the final scores on the training data were in the range of 93.5–95%; for edges between any dynamic image type and static images, the scores were essentially 100%. The output neurons of these networks, if taken as a group, would signal the presence of an edge by activity in any one or more of them.

**Figure 5:**
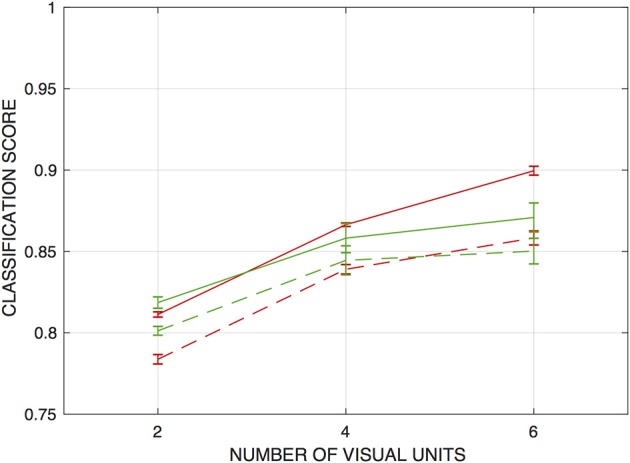
Classification scores for networks trained to detect moving edges (red curves), and both moving and fixed edges (green), as functions of the number of visual units from which inputs are drawn. Scores are the fraction of input patterns correctly assigned. Evaluations take place with the type of data with which each network is trained (i.e., only moving-edge data are presented to networks trained with moving edges, and edges of both types presented to networks trained with both). Solid lines indicate performance on the training sets, and dashed lines performance on holdout sets. Error bars indicate ± one standard deviation (over *n* = 5 independently-trained detection networks in each case).

Networks trained with moving-edge data achieve somewhat lower scores than the fixed-edge networks on both training and test sets, although performance remains well above chance. Results are depicted in Figure 5 with the red data points / curves. Scores for networks drawing from all six visual units averaged around 90% on the training set and 86% on the test set. Discrimination performance falls off more sharply with smaller receptive field size than for the networks trained with stationary edges.

How well are networks trained with *fixed* edges able to discriminate the presence of a *moving* edge, and vice-versa? To answer this question, I evaluated the performance of networks drawing from all six visual units (and trained with the full corpus of training data) on the training set for the opposite edge type. The networks trained with fixed-edge data achieve an average score of only 60.4% correct on the moving-edge data—around 10% better than chance, and some 30% below scores on their own test set. Networks trained with moving-edge data fare no better on the fixed-edge data, scoring just 57.6% on average. Thus, generalization in both cases is poor.

Given these results, it is clearly of interest how well networks trained with *both* types of edges are able to discriminate the presence of either edge type. Results are also shown in Figure 5, and demonstrate that the model can indeed to learn to discriminate the presence of both types of edges at rates much higher than chance —and roughly as well as networks trained with moving edges only.

These networks detect either fixed or moving edges with similar accuracy; for example the mean score on the fixed-edge training data is 86.4% and on the moving-edge training data is 84.9% for the five networks that draw from all six inputs.

### What Resources Are Needed for Effective Discrimination by Image Dynamics?

The effect of the spatial extent of visual field sampled by the edge detection networks on their classification performance was addressed in the prior section. Here, I examine the question of how many of the input components generated by those visual processing units, and how many hidden neurons, are required for effective edge detection, in networks trained with fixed-edge data.

This was tested using the network pruning procedure, by compiling classification scores evaluated as the individual input components or neurons are removed. For the raw vision inputs, this process was carried out for each of the 32 signals from the central four visual units. For the correlator inputs, however, a particular product term was tested and removed for all four of the units—resulting in the deletion of four signals (for internal correlations) or three signals (for inter-unit correlations) per iteration of pruning.

**Figure 6:**
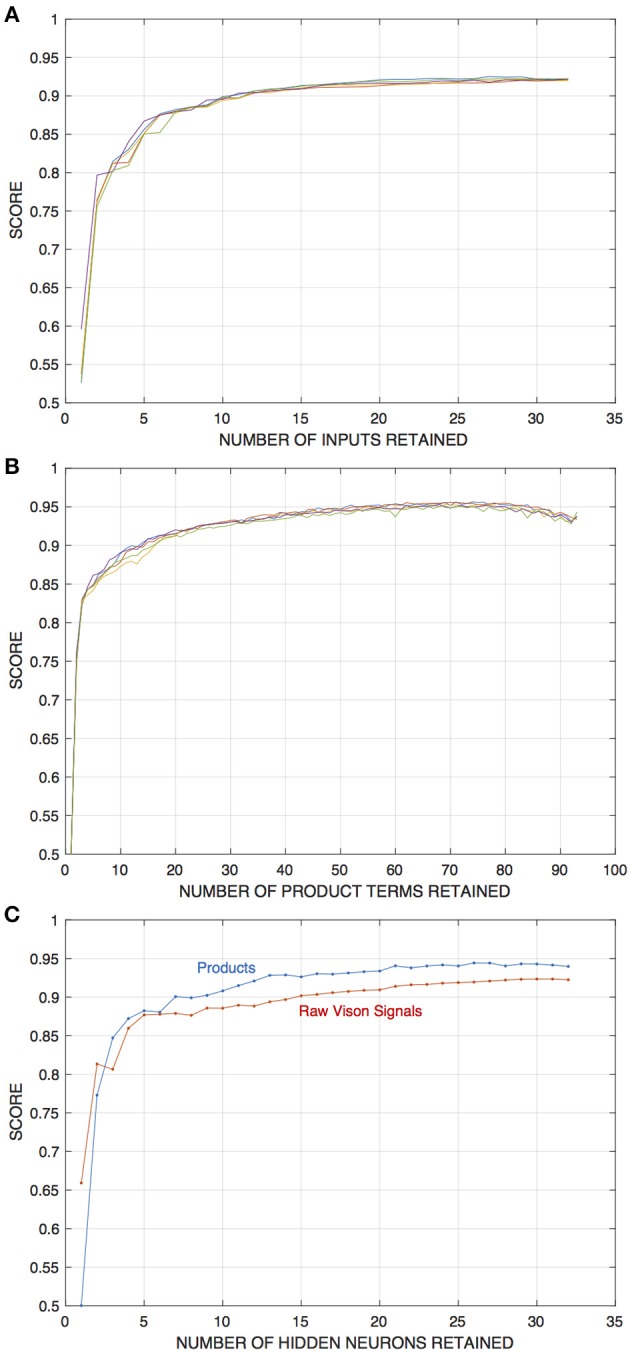
Results of experiments in which either input components or the number of hidden units in edge detection networks are pruned away, in this case from networks that draw inputs from four visual units and are initially trained with 32 hidden units. The training and evaluation dataset is for the fixed-edge case. Pruning and retraining is as described in the text. **(A)** Depicts scores on the training set as a function of the number of input components retained for networks that operate on raw visual signals. **(B)** Shows scores for networks that operate on “correlations” or product signals (In this case, the product terms corresponding to interaction between a given pair of signals are removed for all four visual units at each stage of the pruning process). **(C)** Shows scores as a function of the number of hidden units in the network, for networks with each type of input as indicated.

The results of these experiments indicate that, relative to the baseline numbers, appreciably fewer input components or hidden units are required to achieve edge detection performance well above chance. In either case a significant drop-off in performance occurs only when either number falls below around ten. Figure 6 depicts these results.

Which signals among either the raw vision outputs or the correlations are the most useful for discriminating edges? In order to address this question, I examine the 10 most significant of those signals as determined by the final 10 steps of the pruning process, and catalog those that remain at each step, for each of the five initial network configurations that generated the data in Figure 6. Significance is quantified as follows: each input component is scored according to the final pruning step in which it was present, counting from first to the last (e.g., the final remaining component in a network is assigned a score of ten), and these numbers are averaged over the sample of five different networks. In this average, components that are not present in all of the networks are ranked zero for those in which they did not appear. The results are summarized in Table 1.

**Table 1:** Lists of the 10 most significant raw vision signals (left) and product terms (right) for edge discrimination in individual networks, compiled over five-network samples.

**Raw vision signals**				**Products**			
**Rank**	**Score**	**Output components**	**Occurrences**	**Rank**	**Score**	**Product terms**	**Occurrences**
1	8.6	SUS_ON_D-RightMid	5	1	9.6	SUS_ON_D-Left * SUS_ON_D-Right	5
2a	7.4	SUS_ON_D-LeftMid	4	2	9.4	SUS_OFF_D-Left * SUS_OFF_D-Right	5
2b	7.4	SUS_ON_D-Left	5	3	7.0	SUS_ON_D * SUS_ON_U	5
3	7.2	SUS_OFF_D-Right	5	4	6.4	SUS_OFF_D * SUS_OFF_U	5
4	6.6	SUS_OFF_D-LeftMid	5	5	5.8	SUS_OFF_D-Left * SUS_OFF_U-Right	5
5	6.2	SUS_OFF_D-RightMid	5	6	4.8	SUS_ON_D-Left* SUS_ON_U-Right	5
6	4.4	SUS_OFF_D-Left	5	7	4.0	SUS_ON_U-Left * SUS_ON_D-Right	5
7	3.6	SUS_ON_D-Right	5	8	2.2	SUS_OFF_U-Left * SUS_OFF_D-Right	4
8	1.8	TRN_OFF_D-RightMid	4	9	2.0	SUS_OFF_D * SUS_ON_D	3
9	1.0	TRN_OFF_D-LeftMid	4	10	0.8	SUS_ON_U-Left * SUS_OFF_D-Right	1
10	0.6	TRN_ON_D-RightMid	2	11a	0.6	SUS_OFF_D-Left * SUS_ON_U-Right	1
11	0.2	TRN_ON_D-LeftMid	1	11b	0.6	SUS_OFF_U-Left * SUS_OFF_U-Right	1
				12a	0.4	SUS_OFF_D* SUS_ON_U	1
				12b	0.4	SUS_OFF_D-Left * SUS_ON_D-Right	1
				12c	0.4	SUS_ON_D-Left * SUS_OFF_D-Right	1
				12d	0.4	TRN_OFF_D-Left* SUS_OFF_D-Right	1
				13	0.2	SUS_OFF_D-Left* TRN_OFF_D-Right	1

*Ranks and significance scores are computed as described in the text. “Occurrences” indicates the number of networks in the sample in which an input was among the ten most significant. SUS designates sustained signals; TRN, transient signals; ON, ON pathway; OFF, OFF pathway; U, undelayed signals; D, delayed signals. For the raw vision signals, LeftMid and RightMid designate the units adjacent to the center of the array, and Left and Right indicate the outermost two units. For the product terms, Left and Right designate factors drawn from two neighboring pairs of units. When Left and/or Right do not appear, the factors are drawn from the same unit*.

One observation of interest with the pruned raw-vision-based networks is that *all* ten most significant components in *each* of the five of them are *delayed* signals. The eight delayed ON and OFF sustained components comprise the eight most significant components over the sample. Four delayed transient signals—those for two central visual units—also appear among the inputs these networks, with lower frequencies and significance than any of the sustained signals. In the product-based networks, the delayed signals also outnumber the undelayed signals as factors in the ten most significant input components. In addition, these factors are dominated by sustained signals: transient signals (both delayed) appear in only two products, with minimal significance.

**Figure 7:**
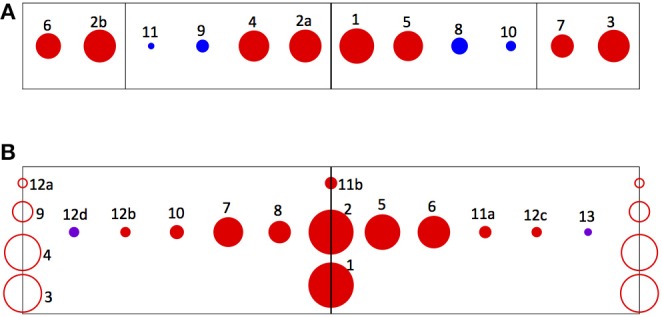
Ideograms depicting the degree of left-right symmetry of the most significant input components for edge detection, as identified in the pruning experiments. Pairs of inputs related by left-right reflections are represented by marker pairs placed symmetrically about the midline. The area of each marker is proportional to the significance of its component, as given in Table 1; the number adjacent to each is its rank, to allow its identification. **(A)** Depicts the most significant raw vision signals. Red markers denote sustained signals and blue, transient signals. The four compartments defined by the vertical lines in the ideogram correspond to the four visual units from which the signals originate, from left to right across the array. **(B)** Depicts the most significant product signals. Red markers denote products between sustained signals and violet, products between one sustained and one transient signal. The markers placed on the centerline represent products that are identical to their own reflections (for example, SUS_ON_D-Left^*^SUS_ON_D-Right). The markers placed on the end lines represent products of signals from individual units, which have no reflections (for example, SUS_ON_D^*^SUS_ON_U from any single unit).

How close is ANN-base edge discrimination able to come to an optimal solution of the general edge detection problem, given the limits of the finite training sample and the learning procedure? While classification scores measure *performance*, the fact that they are <100% may simply reflect the fact that edged and edgeless classes are not perfectly separable based on the signals they induce in the visual model. Another way to address this question is to evaluate the *symmetry* of the most significant input components that are selected by pruning trained networks. This is because a reflective symmetry prevails in the data used to determine the weights: every class of input pattern is represented equally with the class that corresponds to its reflection about the center of the array (e.g., for every visual datum generated by the edged image class [Flicker | Motion Left], there is a datum generated by the [Motion Right | Flicker] class). Consequently, any subset of input components selected for optimal edge discrimination in trained networks would be expected to retain symmetry with respect to such left-right interchange. The degree to which this is true in the pruned networks of Table 1 can be assessed visually in Figure 7. The ideograms in Figure 7 indicate the input components from the ten final pruning stages in the five network sample, using an array of markers in which pairs of inputs related by left-right reflections are represented by marker pairs placed symmetrically about the midline. The area of each of the markers is proportional to the significance of its component. Symmetry prevails approximately, but not perfectly: a reflected input is present for every component admitting a reflection, although the significance scores of the two may differ. The sources of such asymmetry might include both bias in the finite training set, and imperfect learning due to incomplete convergence or convergence on suboptimal minima of the loss function.

## Discussion and Conclusions

The primary conclusion from this study is that it is entirely plausible that neural networks operating on early vision signals can discriminate the presence of edges in visual scenes, and can do so based on relatively general differences in spatiotemporal characteristics of the imagery on either side. Such edges are likely to be the primitive features on which the detection of finite (non-point)-sized objects are based (Aptekar et al., 2017; Keleş and Frye, 2017). Although not based on detailed physiology, the visual signals that were modeled are representative of processing that is believed to occur in the early optic ganglia—and as such have been implicated in other functions as well. The discrimination network itself is a simplified analog of a real neural network, consisting of minimalistic model neurons of the kind used in “artificial neural network” models, but which nonetheless captures some of the most basic features of real neurons, such as the integration of excitatory and inhibitory inputs and a compressive or limiting, non-linear input-output relation.

The model is also primitive geometrically, consisting of a single row of receptors with imagery in which edges are located between the central pair. Eyes of course view two-dimensional projections, in which edges may be oriented in arbitrary directions on the retina. In order to detect edges with different orientations, the corresponding neural machinery would have to be replicated not just once per visual processing column, but for multiple inter-receptor axes between columns. Edges in natural images typically span multiple receptors, which could be exploited to improve the reliability of detection by integrating evidence from multiple sets of visual columns. However, edges projected on a hexagonal ommatidial array may be misaligned with respect to inter-receptor axes by up to 30°, which might be expected to negatively impact detection reliability.

Interestingly, networks trained to detect the presence of fixed edges generalize poorly when the edges are animated—and vice-versa. This suggests the cues based on image dynamics that are used by the fixed-edge networks are significantly disrupted when an edge moves, and conversely that networks trained to detect moving edges rely strongly on cues induced specifically by edge motion. When edges move, luminance discontinuities across them naturally generate transient signals in the neighboring receptors, and these are often quite large compared to variations associated with the internal dynamics of the subimages. Nevertheless, networks trained with both fixed and moving edges can detect either type at rates far better than chance, demonstrating that they are able to draw information from both sorts of cues.

The study suggests that the resources—i.e., the number of primitive signals and the number of neurons—needed to achieve discrimination of edges are realistic, relative to the known neuroanatomy of the insect medulla. The model contains a “hidden layer” of neural analogs that operates on raw signals produced by earlier visual processing, or alternatively on “correlations” or products formed between them. With the fixed edges used for the bulk of the study, pruning experiments showed that on the order of a dozen signals drawn from two columns on either side of an edge are sufficient to give discrimination far above chance (although these signals must be capable of inducing excitation or inhibition, requiring projections or interneurons of each type). Similarly, the number of “hidden neurons” in the model, corresponding to interneurons that extract features from the dynamic signals, may be as few as a dozen or so. Conversely, there are on the order of 50 medullar cell types and over 350 individual cells found within each column of the medulla in *Drosophila*, with some being intrinsic, and some extending across multiple columns (Takemura et al., 2013).

In examining the significance of the input signals to the edge detection network, it is perhaps unsurprising that the transient signals play a less significant role than the sustained signals. The undelayed transient signals in particular are active for very short periods of time, and during extended periods of inactivity provide no information for discrimination. Thus, as might be expected, only delayed transient signals appear among the more significant inputs. However, it is of interest that all the most significant *sustained* signals identified in the study are *also* delayed: why should this be the case? Information for distinguishing moving stimuli is implicit in the responses of spatially-offset visual columns that view the motion, but when both delayed and undelayed signals are present, this allows temporal information to also be conveyed via phase delay. I speculate that the significance of the delayed signals must have to do with their modified time course or harmonic content. The harmonic structure of the sustained signals tends to be quite rich, whereas higher-frequency signal components are suppressed and the signals are “smeared out” when delayed by the lowpass filter. These observations raise the possibility that signal characteristics in the biological system are more suited for edge discrimination than the simple model signals in this study. For example, there are no compressive non-linearities, no filtering effects that suppress higher frequencies (beyond the delay filter), and no adaptation (after the photoreceptors) in the early vision signal chain—whereas these features are common in real neurons and neural networks. The resultant differences in signal characteristics might be expected to influence discrimination capability.

The computations performed by this model can be divided into early vision operations with straightforward interpretations, and ANN operations that are a result of stochastic approximation, and thus tend to be more cryptic in nature. The computations that lead to edge discrimination capability are *distributed* in the ANN, and even though presence or absence of an edge is (arbitrarily) coded by a single “neuron” in the basic network, the networks that were trained on single edge classes had multiple output neurons and showed that a distributed output representation is functionally just as practical. Decades of study of artificial neural networks trained with stochastic learning procedures have shown it is often difficult to interpret their distributed computations and/or internal representations in terms of ultimate network function—and there seems to be little reason to believe that computational neural networks resulting from evolutionary and developmental processes would be any different in this regard. If so, this presents a hurdle for efforts to interpret network function by focusing on cellular-level responses, as revealed by techniques such as electrophysiological recordings or calcium imaging. Such difficulties are evident in efforts to interpret the responses of candidate neurons for figure processing (e.g., Egelhaaf, 1985; Liang et al., 2012) and indeed, recent results obtained from two-photon imaging of such neurons in *Drosophila* give intriguing but difficult-to-interpret results in response to moving object and moving edge stimuli (Keleş and Frye, 2017). A true understanding of the computations that give rise to capabilities such as general edge and object discrimination may well depend on multi-unit experimental neurophysiological techniques that allow the evaluation of *network* function.

## Data Availability Statement

The datasets generated for this study are available on request to the corresponding author.

## Author Contributions

PS designed the study, wrote the software, conducted and analyzed the results of the experiments, and wrote the manuscript.

### Conflict of Interest

The author declares that the research was conducted in the absence of any commercial or financial relationships that could be construed as a potential conflict of interest.

## Supplementary Material

Supplementary Movie 1 Example segment of dynamic image data, for a scenario with a fixed edge that separates leftward motion in the left hemifield from flicker in the right. The abscissa represents luminance and the ordinate, position in the one-dimensional visual field. The blue trace shows the original high-resolution image data, and the red shows the blurred and downsampled data, with markers indicating the eight input data presented to the edge detection model.

Supplementary Movie 2 Example segment of dynamic image data, for a scenario with moving edges separating static regions and regions of theta motion (motion of texture in the direction opposite to motion of the edges). The abscissa represents luminance and the ordinate, position in the one-dimensional visual field. The blue trace shows the original high-resolution image data, and the red trace shows the blurred and downsampled data, with markers indicating the eight input data presented to the edge detection model.
